# A longitudinal observational retrospective study on risk factors and predictive model of PICC associated thrombosis in cancer patients

**DOI:** 10.1038/s41598-020-67038-x

**Published:** 2020-06-22

**Authors:** Xiaomin Song, Hong Lu, Fang Chen, Zuowei Bao, Shanquan Li, Siqin Li, Yinghua Peng, Qiao Liu, Xiaohui Chen, Jingzhen Li, Weimin Zhang

**Affiliations:** 1 Department of Radiology, National Cancer Center/National Clinical Research Center for Cancer/Cancer Hospital & Shenzhen Hospital, Chinese Academy of Medical Sciences and Peking Union Medical College, ShenZhen, 518116 China; 2 Administrative Department of Nurse, National Cancer Center/National Clinical Research Center for Cancer/Cancer Hospital & Shenzhen Hospital, Chinese Academy of Medical Sciences and Peking Union Medical College, ShenZhen, 518116 China; 3 Department of Ultrasound, National Cancer Center/National Clinical Research Center for Cancer/Cancer Hospital & Shenzhen Hospital, Chinese Academy of Medical Sciences and Peking Union Medical College, ShenZhen, 518116 China; 4 Department of Ultrasound, The third people’s Hospital of ChangZhou, JiangSu Province, 213001 China; 5 Department of Intravenous lab, National Cancer Center/National Clinical Research Center for Cancer/Cancer Hospital & Shenzhen Hospital, Chinese Academy of Medical Sciences and Peking Union Medical College, ShenZhen, 518116 China

**Keywords:** Cancer imaging, Risk factors

## Abstract

To analyze the incidence of PICC associated venous thrombosis. To predict the risk factors of thrombosis. To validate the best predictive model in predicting PICC associated thrombosis. Consecutive oncology cases in 341 who initially naive intended to be inserted central catheter for chemotherapy, were recruited to our dedicated intravenous lab. All patients used the same gauge catheter, Primary endpoint was thrombosis formation, the secondary endpoint was infusion termination without thrombosis. Two patients were excluded. 339 patients were divided into thrombosis group in 59 (17.4%) and non-thrombosis Group in 280 (82.6%), retrospectively. Tumor, Sex, Age, Weight, Height, BMI, BSA, PS, WBC, BPC, PT, D-dimer, APTT, FIB, Smoking history, Location, Catheter length, Ratio and Number as independent variables were analyzed by Fisher’s scoring, then Logistic risk regression, ROC analysis and nomogram was introduced. Total incidence was 17.4%. Venous mural thrombosis in 2 (3.4%), “fibrin sleeves” in 55 (93.2%), mixed thrombus in 2 (3.4%), symptomatic thrombosis in 2 (3.4%), asymptomatic thrombosis in 57 (96.6%), respectively. Height (χ² = 4.48, *P* = 0.03), D-dimer (χ² = 37.81, *P* < 0.001), Location (χ² = 7.56, *P* = 0.006), Number (χ² = 43.64, *P* < 0.001), Ratio (χ² = 4.38, *P* = 0.04), and PS (χ² = 58.78, *P* < 0.001), were statistical differences between the two groups analyzed by Fisher’s scoring. Logistic risk regression revealed that Height (β = −0.05, *HR* = 0.95, 95%CI: 0.911–0.997, *P* = 0.038), PS (β = 1.07, *HR* = 2.91, 95%*CI*: 1.98–4.27, *P* < 0.001), D-dimer (β0.11, *HR* = 1.12, 95%*CI*: 1.045–1.200, *P* < 0.001), Number (β = 0.87, *HR* = 2.38, 95% *CI*: 1.619–3.512, *P* < 0.001) was independently associated with PICC associated thrombosis. The best prediction model, D-dimer + Number as a novel co-variable was validated in diagnosing PICC associated thrombosis before PICC. Our research revealed that variables PS, Number, D-dimer and Height were risk factors for PICC associated thrombosis, which were slightly associated with PICC related thrombosis, in which, PS was the relatively strongest independent risk factor of PICC related thrombosis.

## Introduction

Due to the toxicity of chemotherapy to the vascular wall, it is essential to establish a reliable, relatively long-term central venous access for cancer patients who required intravenous chemotherapy. Peripherally Inserted Central Catheter (PICC) was widely used in clinical scenarios because of its easily being performed, safety, long indwelling time, easy care and maintenance, less discomfort and low risk of infection. Most importantly, PICC, as an effective alternative to central venous catheter (CVC) could effectively avoid CVC related critical iatrogenic pneumothorax and gas embolism complications^1–4^.

However, multiple studies have confirmed high overall incidence (up to 30–40%) of PICC catheter related complications, including deep vein thrombosis (DVT), infection, fibrin sheath, superficial phlebitis, catheter prolapse, catheter displacement and blockage^5,6^, among which, indwelling catheter vein and/or fibrin-sheath-shape catheter thrombosis prevails^7,8^.

This paper summarized the incidence and analyzed the risk factors for PICC associated thrombosis in 339 cancer patients who received PICC placement, retrospectively. Based on the risk factors, a model was built and validated to predict the risk for PICC associated thrombosis.

## Materials and Methods

### Study design

**Figure 1:**
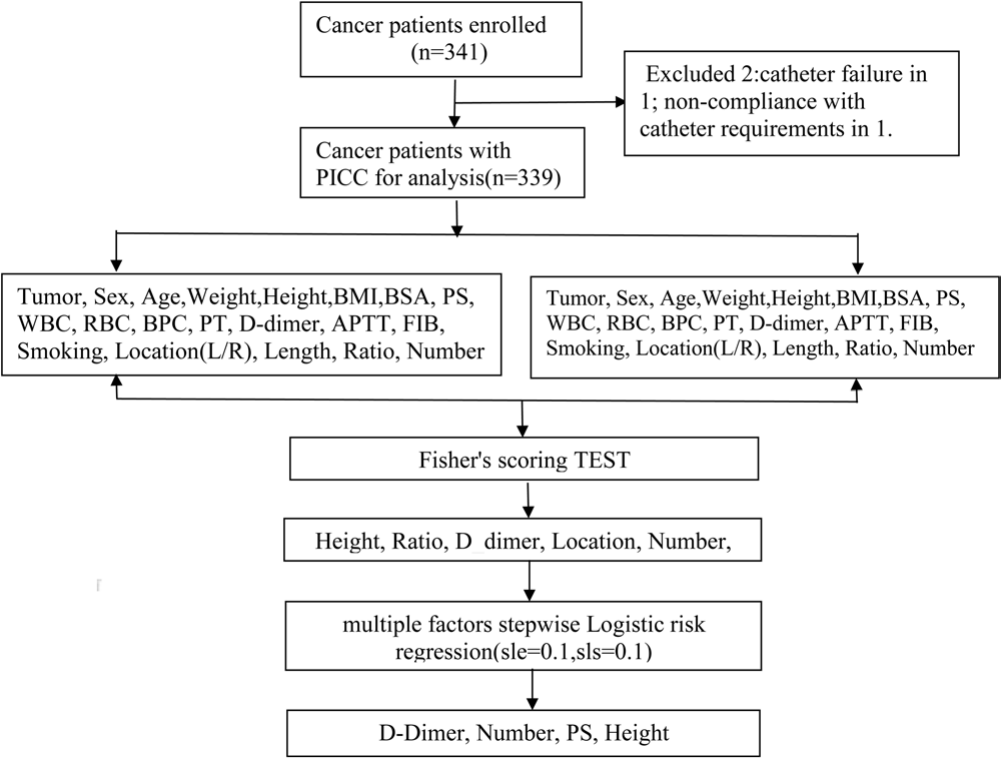
The patient flowchart.

The manuscript on reporting results of clinical trial conforms to CONSORT 2010 guidelines. Since January 2018, in 10 months, based on the medical records, 341 cancer patients who initially naive intended to subject to PICC placement in our intravenous catheter center were included in this retrospective study with 1 case excluded for placement failure, 1 case for non-compliance (Fig. 1). Primary endpoint was thrombosis formation detected by ultrasound examination, the secondary main endpoint was IV infusion termination without PICC relative thrombosis formation. During this period of time, patients with thrombosis detected by ultrasound examination or during the removal of PICC line were assigned to thrombosis group, otherwise to the non-thrombosis group. Because of the retrospective nature of the study, the study protocol was approved by the Hospital Ethics Committee (National Cancer Center/National Clinical Research Center for Cancer/Cancer Hospital & Shenzhen Hospital, Chinese Academy of Medical Sciences and Peking Union Medical College, ShenZhen, 518116, China), which waived the requirement for informed consent as the data were analyzed anonymously and the patient privacy was protected. This study was carried out in accordance with Good Clinical Practice (GCP) guidelines and the Declaration of Helsinki.

### Materials

Poly RadPICC® 4 Fr Single-Lumen Catheter, Basic Tray with 70 cm Stainless Steel Guidewire.(C. R. Bard, Inc. USA).

Volusion E8 ultrasound systems. 11 L Transducer, Vascular application (GE, Healthcare, Austria GmbH&Co OG).

### Methods

All the procedures of PICC insertion were performed by experienced certified nurses under the standard protocol and guideline in our intravenous catheter center. All ultra-sound images were explained by two senior ultrasound experts. From the procedure record and later medical records, we extracted following information, including: (1) catheter length on insertion; (2) any complications during the procedure (bleeding, nerve injury, irregular heartbeat, damage to veins in the arm, blood clots, infection, blocked or broken PICC line, etc); (3) insertion site and venous access site; (4). the vessel-to-catheter Ratio in diameter; (5). puncture times; (6). L/R limb.

### Clinical information acquisition

From the medical records, we acquired basic clinical characteristics for each patient, including male, 143(42.2%), mean age (56.8 ± 13.2yo) female, 196(57.8%), mean age (52.9 ± 14.8yo); cancer type, solid tumors in 296 (87.3%), haematopoietic tumor in 43(12.7%); smoking history (more than 20 years, more than 360 packets per year) in 38(11.2%). Weight, Height, Body Mass Index (BMI), Body Surface Area (BSA); Patient’s performance status (ECOG)(PS), baseline laboratory test results, and other thrombosis related history. In specific, for baseline laboratory test, we obtained following information including: (1) White Blood Count (WBC); (2) D-dimer; (3) Prothrombin Time (PT); (4) Activated Partial Thromboplastin Time (APTT); (5) Blood Platelet Count (BPC); (6) Fibrinogen (FIB). In addition, history of vascular access, prior surgical procedures, localized trauma at insertion site, history of previous catheter related and non-catheter-related thrombosis, and previous anticoagulant therapies were also extracted from the medical record. All patients underwent PICC for the first time, including head and neck cancer, lung cancer, breast cancer, ovarian cancer, and lymphoma, etc.

The basilica vein, approximately 4–10 cm above the elbow, was choosed as the preferred venipuncture site in 90% and brachial vein in 10% as alternative target vessel due to easier accessible than basilic vein. L/R limb was randomized. After the skin disinfection, the Drapes covered, inserted acuductor as soon as the puncture succeeded, the bevel of the needle should be directed to the heart direction, loosen the tourniquet, extracted the needle from the vein while inserted the catheter and the guide wire along the acuductor, extracted the guide wire and the acuductor in turns from the catheter. All patients used the same gauge catheter. The catheter tips were located in the proximal superior vena cava (SVC), which guilded by real time ECG monitoring during the operation and verified by postoperative X-ray Exam.

With high frequency high definition ultrasound system, scanned from puncture point site, along the vascular pathway through the basilic vein, brachial vein, and axillary vein, to proximal subclavian vein. The PICC catheter and indwelling vein were scanned longitudinally and transversely, applied the appropriate transverse pressure to the vessel to differentiate thromboses from blood stasis, while the patient was instructed to valsalva maneuver.

### Evaluation before and after PICC placement

Before PICC placement, each patient underwent ultrasonograpy (US) assessment, which focused on the route of the target vessel and its morphology. Specifically, presence of (1) winding or irregular pathway, (2) bifurcations or sudden bending of the vein, or (3) the evidence of valves was recorded in a structural ultrasound report.

Right after PICC placement, all the patients received a chest X-ray. From the X-ray report, we acquired information as follows: (1) potential malposition and anatomical level of catheter tip; (2) trace and documentation of the position of the line. Two weeks after PICC placement, all the patients routinely underwent ultrasound evaluation. From the ultrasound report, we obtained information regarding the presence of PICC associated thrombosis or not.

### Classification of thrombosis

Based on the ultrasound findings, positive thromboses were classified into three types, including: (1) vein mural thrombus, defined as thrombosis attached to the targeted vein mural; (2) catheter-adherent-fibrin-sleeves thrombus, defined as thrombosis attached to the exterior surface of catheter; (3) mixed thrombus, defined as vein mural thrombus with catheter-adherent-fibrin-sleeves thrombus.

Based on whether symptoms or signs were developed, thromboses were divided into symptomatic and asymptomatic thrombosis. PICC related symptoms or signs included infection, local pain, edema, dyspnea, and cardiac failure.

### Statistical analysis

Continuous variables were presented as mean ± standard deviation. Categorical data were presented as proportions. First, the classification data parameters for quantitative conversion, SAS V8 (SAS, CARY, NC, USA) for each measurement parameter, Mean ± standard deviation test, then Fisher’s scoring inspection. To analyze the potential risk of PICC associated venous thrombosis between two groups. The potential risk factors identified by Fisher’s exact test were fed into Multivariate regression analysis, and using stepwise regression analysis (sle = 0.1, sls = 0.1), the independent risk factors of PICC related venous thrombosis were determined, A two-tailed level of significance of 0.05 was applied. For ROC analysis, Non-parametric hypothesis, one-tailed level of significance of 0.05 was applied. Each of risk variables or the randomized combined risk co-variables as a novel co-variable was analyzed by the ROC curve analysis of the SPSS software (IBM SPSS Statistics 26.0). The sensitivity, False Positive Rate (FPR) and Overall Model Quality (OMQ) of each risk co-variable were obtained. After assigning weight to each variable, a signature combining two, three, four variables as individual novel variable was fed into the ROC curve analysis of the SPSS software. In terms of risk regression coefficient of PS, Number and D-dimer, we used the R language (R version 4.0.0 .2020-04-24) to make the nomogram for risk factors.

### Ethics approval and consent to participate

Because of the retrospective nature of the study, the study protocol was approved by by the Hospital Ethics Committee (National Cancer Center/National Clinical Research Center for Cancer/Cancer Hospital & Shenzhen Hospital, Chinese Academy of Medical Sciences and Peking Union Medical College, ShenZhen, 518116, China), which waived the requirement for informed consent as the data were analyzed anonymously and the patient privacy was protected. This study was carried out in accordance with Good Clinical Practice (GCP) guidelines and the Declaration of Helsinki.

## Results

### Characteristics of the patients

Figure 1 presents the patient flowchart. During this time period, 339 cancer patients received PICC line placement. Among all the included cancer patients, 59 patients (17.4%) who developed PICC associated thrombosis during the PICC line placement were assigned into thrombosis group, while the remaining 280 patients (82.6%) were assigned to the non-thrombosis group, respectively. Specifically, fibrin-sleeves thrombosis was the most common type of PICC associated thrombosis accounting for 93.2% (55/59) with venous mural thrombosis in 2 cases (3.4%), and mixed thrombus in 2 cases (3.4%). In terms of PICC associated symptoms and signs, only 2 (2/59) thrombosis cases developed local infectious symptoms, with 57 (57/59) asymptomatic thrombosis.

### The potential risks and multivariate analysis

Table 1 presents the potential risks of PICC associated thrombosis in 339 analyzed by Fisher’s exact test. The Height (χ² = 4.48, *P* = 0.03), Ratio (χ² = 4.38, *P* = 0.04), D-dimer (χ² = 37.81, *P* < 0.001), Location (χ² = 7.56, *P* = 0.01), Number (χ² = 43.64, *P* < 0.001), PS (χ² = 58.78, *P* < 0.001), were statistical significant differences, in which, the significance between the two groups, D-dimer, PS and Puncture times were most pronounced. The average observed FIB value in thrombosis group (4.46 ± 1.57 g/L) and no-thrombosis group (4.50 ± 1.48 g/L) were significantly higher than that of the normal reference FIB (2.0–4.0 g/L), and average observed WBC (11.63 ± 34.76 10^9 /l) in the thrombosis group was also significantly higher than the normal WBC reference value (3.5–9.5 10^9/l). D-dimer value in the thrombosis (5.84 ± 6.62 μg/ml) and in the no-thrombosis group (2.00 ± 3.38 μg/ml) were considerably higher than that of the normal reference (0–0.5 μg/ml).

**Table 1:** Demographic and clinical characteristics of the patients.

Risk factor	Thrombus group		non-thrombus group		Chi-Square	*P*
	Mean	Standard Deviation	Mean	Standard Deviation		
Solid	0.95	0.22	0.86	0.35	3.72	0.05
Sex	1.39	0.49	1.43	0.50	0.30	0.58
Age	56.76	13.21	52.95	14.57	3.43	0.06
Weight	54.92	12.06	57.53	11.01	2.64	0.10
Height	159.46	5.91	161.75	7.83	4.48	0.03
BMI	21.66	4.51	21.89	3.59	0.19	0.66
BSA	1.56	0.17	1.60	0.17	3.12	0.08
PS	1.49	1.12	0.52	0.72	8.78	<0.0001
WBC	11.63	34.76	8.45	20.60	0.89	0.35
RBC	3.98	0.77	4.09	0.73	1.23	0.27
BPC	247.59	115.21	264.90	117.05	1.07	0.30
PT	13.61	2.13	14.18	12.65	0.12	0.73
D-dimer	5.84	6.62	2.00	3.38	37.81	<0.0001
APTT	36.58	7.90	35.90	8.68	0.32	0.57
FIB	4.46	1.57	4.50	1.48	0.03	0.86
Smoking	0.12	0.33	0.11	0.31	0.03	0.86
Location	1.58	0.50	1.38	0.49	7.56	0.006
Length	39.46	3.13	39.78	4.51	0.28	0.60
Ratio	0.98	5.17	0.33	0.06	4.38	0.04
Number	2.14	1.53	1.27	0.63	43.64	<0.0001

Table 2 presents the multivariate analysis for the risks of PICC associated thrombosis. Results revealed that D-dimer (β0.11, HR = 1.12, 95%CI: 1.045–1.200, *P* < 0.001), Number (β0.87, HR = 2.38, 95%CI: 1.619–3.512, *P* < 0.01), PS (β1.07, HR = 2.91, 95%CI: 1.978–4.274, *P* < 0.001), Height (β-0.05, HR = 0.95, 95% CI: 0.911–0.997, *P* = 0.04) were independently associated with PICC associated thrombosis, Location (β0.14, HR = 1.15, 95%CI: 0.877–1.495, *P* = 0.32) was no-independently associated with PICC associated thrombosis Height (β-0.05) was negatively correlated with PICC associated thrombosis. D-dimer (β0.11, HR = 1.12, 95%CI: 1.045–1.200, *P* < 0.001), Puncture times (β0.87, HR = 2.38, 95%CI: 1.619–3.512, *P* < 0.001) were the two key factors in preventing PICC related thrombosis.

**Table 2:** The multivariate analysis for the risk of PICC associated thrombosis (multiple factors stepwise Logistic risk regression, sle = 0.1, sls = 0.1).

Risk Factor	standardized regression coefficient	chi-square	*P*	standard estimate	odd ratio estimates point estimate	95% wald confidence limits
Height	−0.05	4.29	0.04	−0.20	0.95	0.911–0.997
PS	1.07	29.47	0.0001	0.52	2.91	1.978–4.274
D-dimer	0.11	10.33	0.0013	0.27	1.12	1.045–1.200
Location	0.14	0.99	0.32	0.16	1.15	0.877–1.495
Number	0.87	19.34	0.0001	0.44	2.38	1.619–3.512

Location (χ² = 7.56, *P* < 0.01) and Ratio (χ² = 4.38, *P* = 0.04) inspected by Fisher’s exact test manifested significantly between the two groups, and right upper limb superficial vein as PICC catheter preferential location. Although there were differences analyzed by univariate analysis in Location and Ratio between the thrombosis group and the non-thrombosis group, by which, the correlation between the Location, Ratio and thrombosis can be in-distinguished, insignificant correlation between Location (β0.14, HR = 1.15, 95% CI: 0.877–1.495, *P* = 0.32), Ratio and Thrombosis was verified by multivariate regression analysis.

### ROC curve

Variable weight assignment: PS > 1, PS weight assigned to 4, while PS = 0, 1, PS weight assigned to 1. D-dimer > 0.5050 μg/ml, d-dimer weight assigned to 3, while d-dimer < = 0.5050 μg/ml, d-dimer assigned to 1.

Height >158.5 cm, Height assigned to 1, while height < = 158.5 cm, height assigned to 2.

Puncture times >1, Number weight assigned to 3, while puncture times = 1, number weight assigned to 1.

Selected left upper limb as location, location assigned to 2, while right upper limb as location, location assigned to 1.

**Figure 2:**
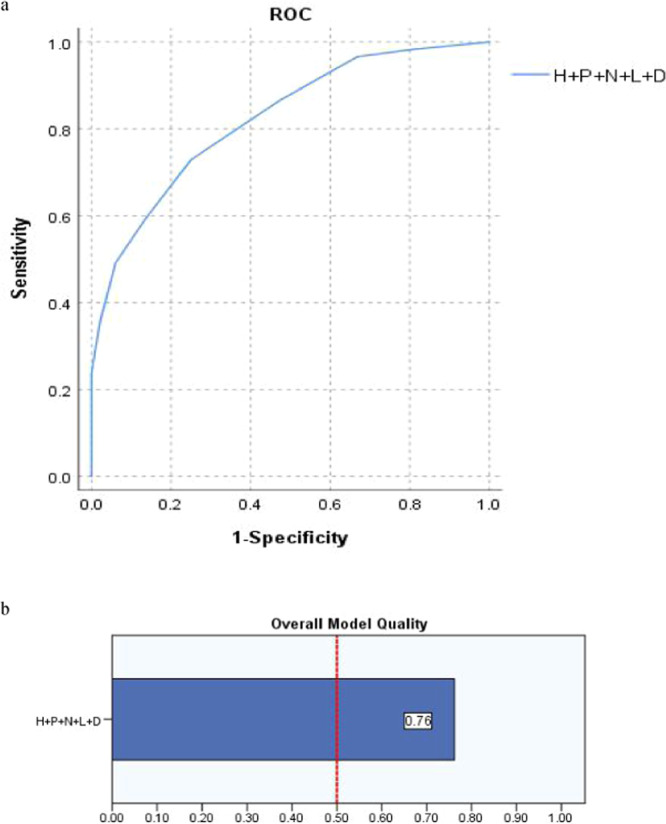
(**a,b**) The sensitivity and FPR of Height + PS + D-dimer + Location + Number (H + P + D + L + N) as new co-variable in predicting PICC associated thrombus by ROC and the overall model quality (OMQ) is 0.76 > 0.5.

Table 3 and Fig. 2(a,b) present, after catheterization, all of the five variables Height + PS + D-dimer + Location + Number (H + P + D + L + N) were included as new co-variable, with cutoff value (8.5scores), the sensitivity (72.9%), False Positive Rate (25.0%), Youden index (0.4788) respectively, predicting PICC related thrombosis was not significantly increased, and the misjudgment rate was also not significantly decreased, which should be the optimized parameter.

**Table 3:** Comparison of Area Under Curve (AUC) and overall model quality (OMQ): 4 or 5 randomized variables as a novel co-variable (Variable weight assignment).

Variables	Cutoff	OMQ	AUC	S.e.m	*P*	95% CI	Sensitivity	1-Specificity
H + P + D + L + N	8.5	0.76	0.822	0.031	0.000	0.762–0.883	0.729	0.250
P + D + L + N	7.5	0.76	0.821	0.031	0.000	0.759–0.882	0.644	0.171
H + P + D + L	6.5	0.72	0.783	0.032	0.000	0.720–0.846	0.763	0.386
H + P + D + N	7.5	0.75	0.812	0.032	0.000	0.749–0.875	0.749	0.644
H + D + L + N	6.5	0.75	0.805	0.030	0.000	0.747–0.863	0.847	0.407
H + L + N + P	6.5	0.71	0.784	0.036	0.000	0.712–0.855	0.644	0.232

**Figure 3:**
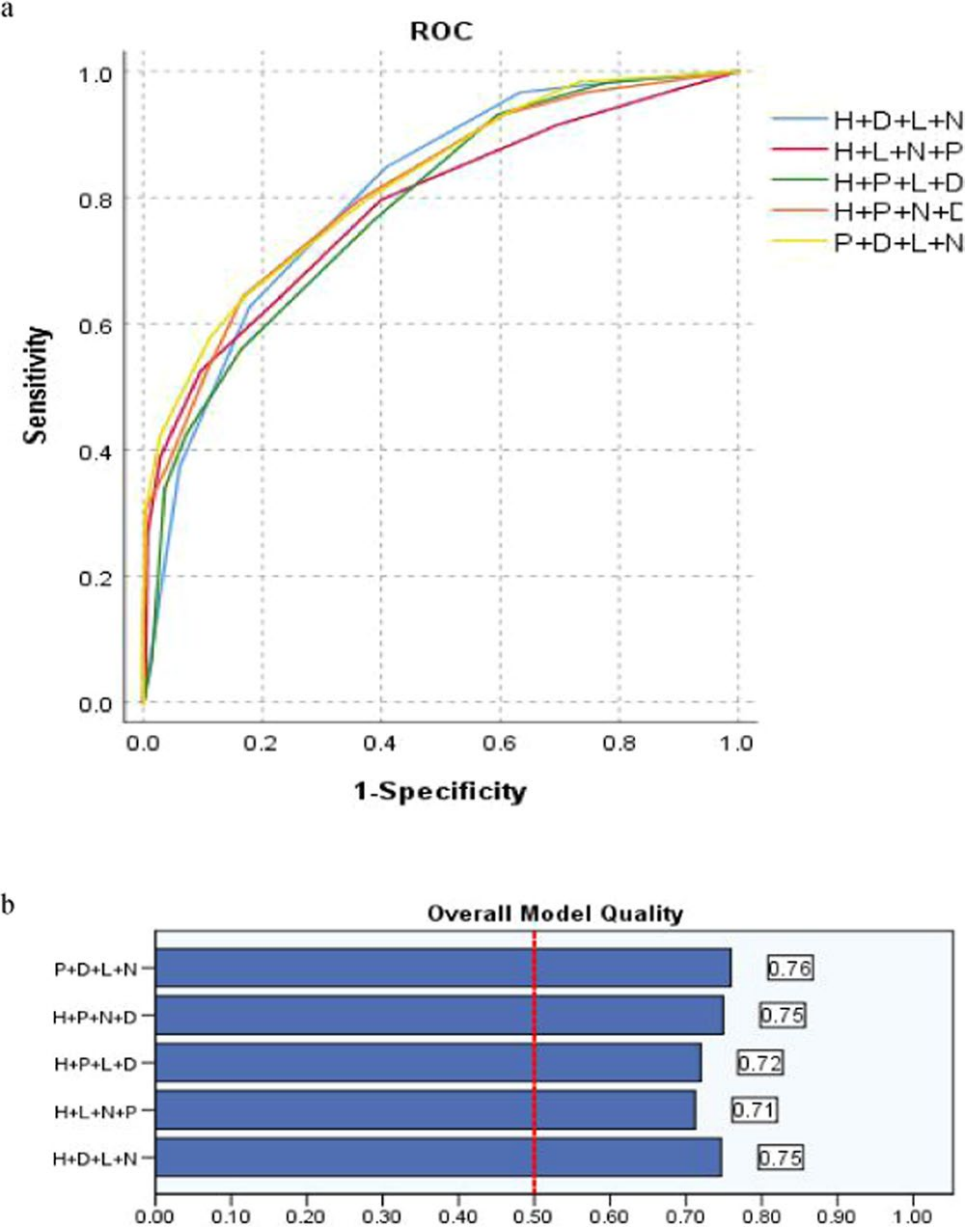
(**a,b**) The sensitivity and FPR of randomized 4 weight variables as new co-variable in predicting PICC associated thrombus by ROC and the overall model quality (OMQ).

Table 3 and Fig. 3(a,b) present, four randomized variables were included as new co-variable, compared with fives, the H + D + L + N as new co-variable should be the most sensitivity (84.7%), Youden index (0.440) and the P + D + L + N should be the least FPR (17.1%), Youden index (0.473) in the model of predicting PICC related thrombus, wheres lack of variable PS or additional Height, the FPR of the model will increased. Four randomized variables as a new co-variable, the sensitivity in predicting PICC related thrombosis was not significantly increased, the misjudgment rate was slightly increased.

**Figure 4:**
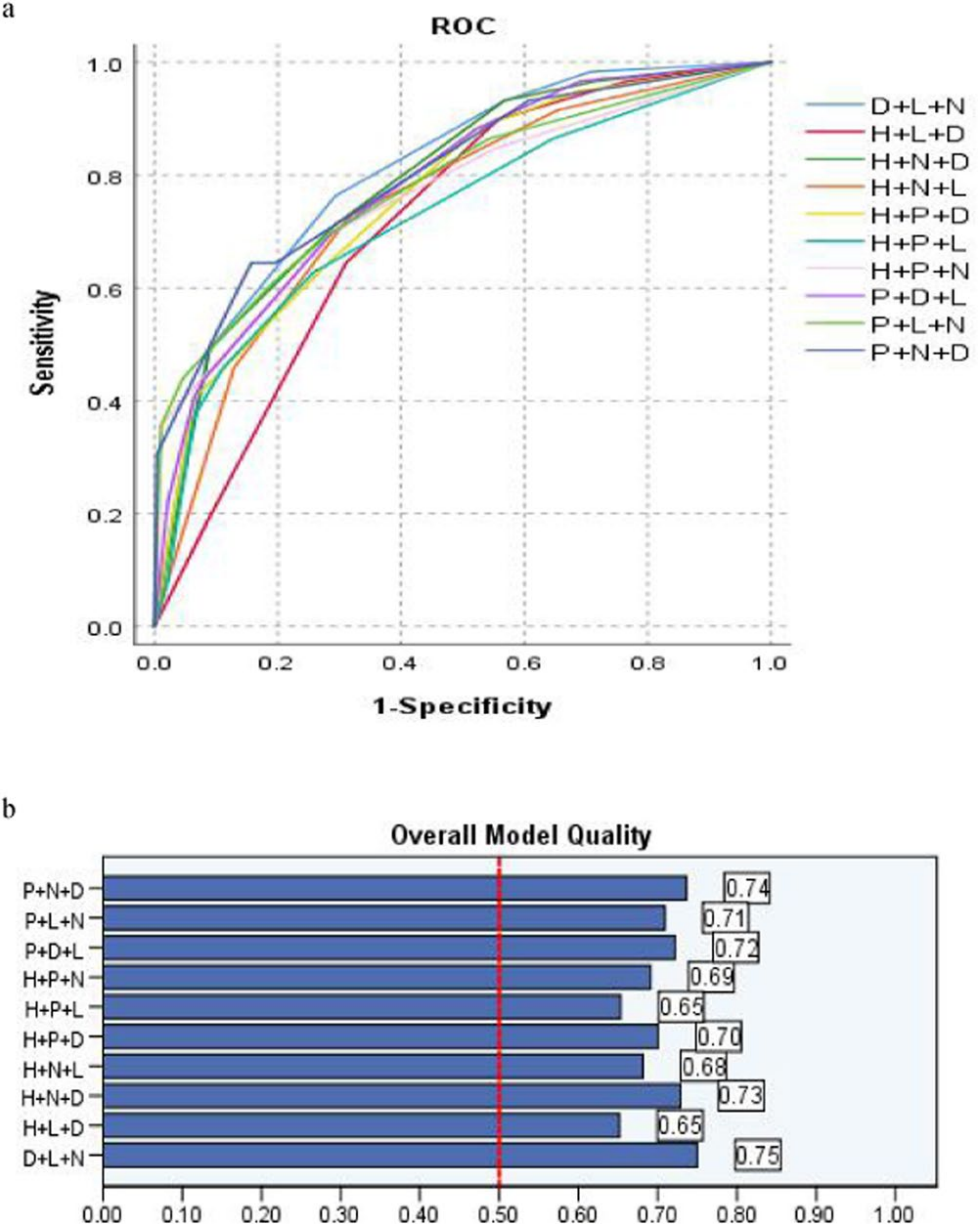
(**a,b**) The sensitivity and FPR of randomized 3 weight variables as new co-variable in predicting PICC associated thrombus by ROC and the overall model quality (OMQ).

Table 4 and Fig. 4(a,b) present, if variable Number (N) or Location (L) were not taken into account before catheterization, Height + PS + D-dimer (H + P + D) to predict PICC associated thrombosis should be the optimized parameter, with cutoff value (5.5scores), the sensitivity (64.4%), False Positive Rate (28.2%) and Youden index (0.3619), respectively, while Puncture + Number + D-dimer (P + N + D) (FPR, 19.6%),

**Table 4:** Comparison of Area Under Curve (AUC) and overall model quality (OMQ): 3 randomized variables as a novel co-variable.

Variables	Cutoff	OMQ	AUC	S.e.m	*P*	95% CI	Sensitivity	1-Specificity
P + D + L	4.5	0.72	0.785	0.032	0.000	0.722–0.848	0.695	0.282
P + L + N	4.5	0.71	0.781	0.037	0.000	0.709–0.853	0.695	0.282
D + L + N	5.5	0.75	0.808	0.030	0.000	0.750–0.866	0.763	0.293
H + P + L	4.5	0.65	0.729	0.039	0.000	0.653–0.806	0.627	0.257
H + P + D	5.5	0.70	0.766	0.034	0.000	0.700–0.832	0.644	0.282
H + N + D	5.5	0.73	0.791	0.032	0.000	0.729–0.852	0.712	0.293
P + N + D	5.5	0.74	0.802	0.034	0.000	0.736–0.868	0.644	0.196
H + P + N	4.5	0.69	0.765	0.038	0.000	0.691–0.839	0.695	0.282
H + N + L	4.5	0.68	0.750	0.035	0.000	0.681–0.818	0.729	0.318
H + L + D	5.5	0.65	0.716	0.033	0.000	0.652–0.787	0.644	0.311

Youden index (0.448) was the lowest one in misdiagnosing PICC related thrombosis.

**Figure 5:**
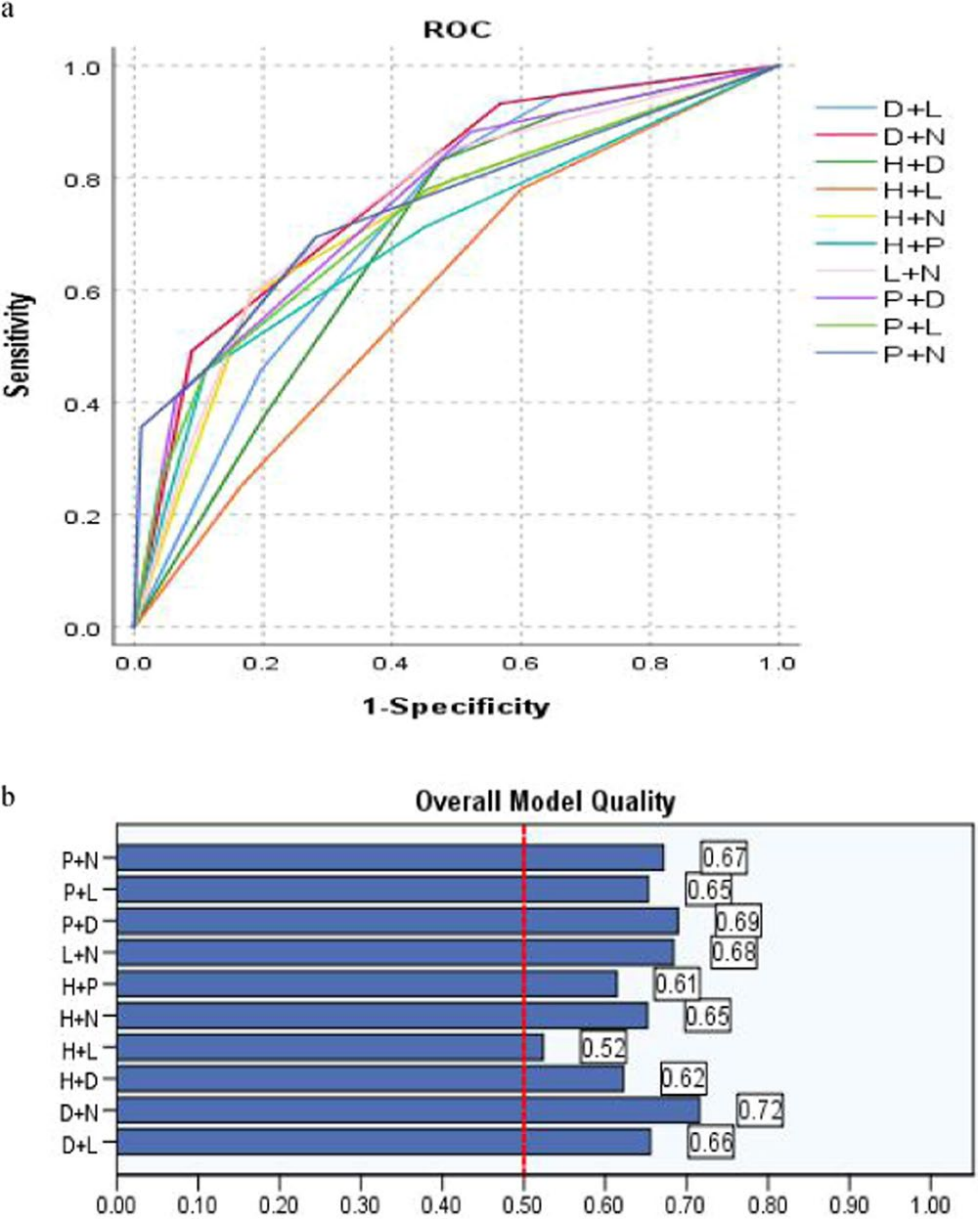
(**a,b**) The sensitivity and FPR of randomized 2 weight variables as new co-variable in predicting PICC associated thrombus by ROC and the overall model quality (OMQ).

Table 5 and Fig. 5(a,b) present, D-dimer + Number as a novel co-variable with a cutoff value (3scores), the sensitivity (93.2%), False Positive Rate (58.5%), Area Under Curve (AUC) (0.780 ± 0.033) and Overall Model Quality (OMQ) (0.72 > 0.5) was validated in diagnosing PICC associated thrombosis before PICC in cancer patients.

**Table 5:** Comparison of Area Under Curve (AUC) and overall model quality (OMQ): 2 randomized variables as a novel co-variable.

Variables	Cutoff	OMQ	AUC	S.e.m	*P*	95% CI	Sensitivity	1-Specificity
P + D	3	0.69	0.758	0.035	0.000	0.689–0.827	0.881	0.521
P + L	2.5	0.65	0.729	0.039	0.000	0.653–0.805	0.780	0.454
P + N	3	0.67	0.750	0.040	0.000	0.672–0.828	0.695	0.282
D + L	3.5	0.66	0.720	0.033	0.000	0.656–0.785	0.831	0.475
D + N	3	0.72	0.780	0.033	0.000	0.716–0.844	0.932	0.585
L + N	2.5	0.68	0.753	0.035	0.000	0.684–0.822	0.847	0.479
H + P	2.5	0.61	0.695	0.041	0.000	0.614–0.776	0.712	0.450
H + N	2.5	0.65	0.726	0.038	0.000	0.652–0.801	0.797	0.493
H + L	2.5	0.52	0.601	0.039	0.010	0.524–0.678	0.780	0.600
H + D	3.5	0.62	0.690	0.034	0.000	0.622–0.757	0.831	0.475

With the increase of variables, the sensitivity of predicting PICC related thrombosis increased gradually, but the misdiagnosis rate also increased significantly.

**Figure 6:**
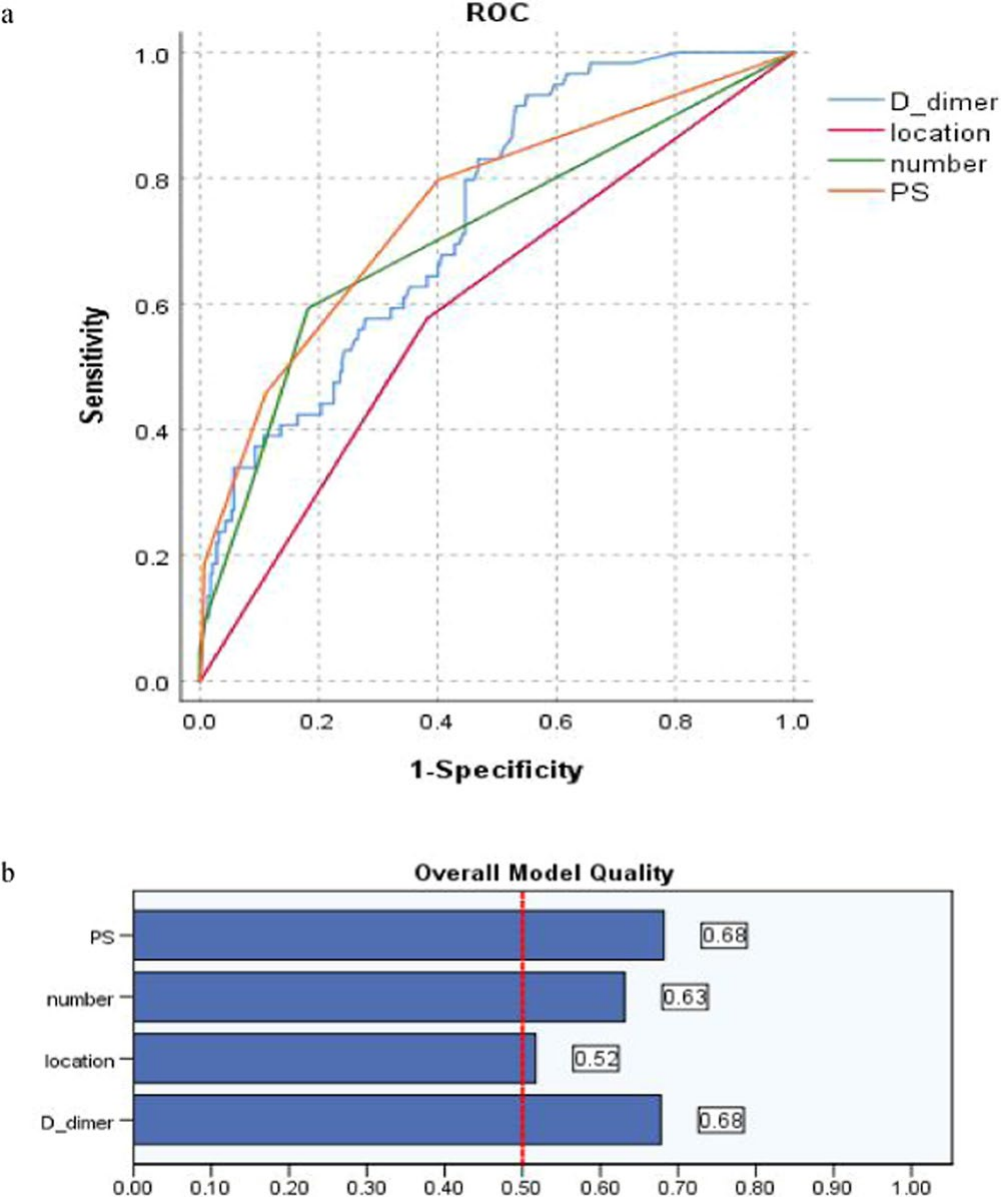
(**a,b**) The sensitivity and FPR of randomized 1 weight variable as new co-variable in predicting PICC associated thrombus by ROC and the overall model quality (OMQ).

Table 6 and Fig. 6(a,b) present, as independent risk variable derived from Fisher’s exact test, PS with the sensitivity (79.7%), FPR (40%), AUC (0.754, 95% CI 0.682-0.827) and Youden index (0.3966) when the cutoff value was more than 1, while the D-dimer with the sensitivity (83.1%), FPR (48.9%), AUC (0.735, 95%CI 0.671–0.798), when cutoff value was more than 0.5050 μg/ml, validated the identical risk results between the regression and the ROC analysis. The most sensitive variable should be d-dimer (sensitivity 83.1%) if a variable were selected for PICC associated thrombus prediction, but the misdiagnosis rate be high (1-specificity 48.9%). Number was less sensitive in predicting PICC associated thrombosis (sensitivity 59.3%), the misdiagnosis rate was also lower (FPR, 18.2%), Youden index (0.4111).

**Table 6:** Comparison of Area Under Curve (AUC) and overall model quality (OMQ): a variable.

Variables	Cutoff	OMQ	AUC	S.e.m	*P*	95% CI	Sensitivity	1-Specificity
Height	158.5	0.52	0.409	0.036	0.028	0.338–0.480	0.614	0.542
PS	1	0.68	0.754	0.037	0.000	0.682–0.827	0.797	0.400
D-dimer	0.5050	0.68	0.735	0.032	0.000	0.671–0.798	0.831	0.489
Location	—	0.52	0.597	0.041	0.019	0.517–0.677	—	—
Number	1	0.63	0.711	0.041	0.000	0.631–0.791	0.593	0.182

**Figure 7:**
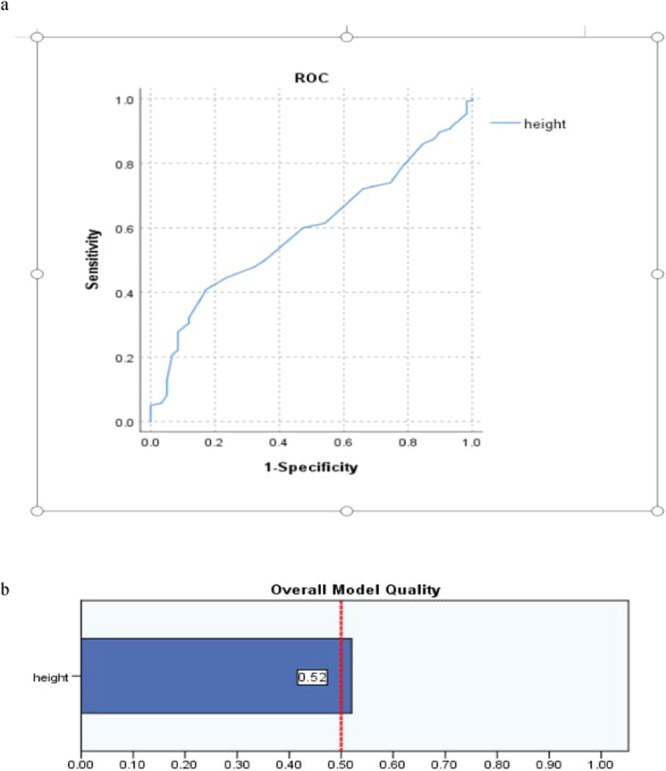
(**a,b**) The sensitivity and FPR of variable Height as new variable in predicting PICC associated thrombus by ROC and the overall model quality (OMQ), specified dependent variable (thrombosis): 0.

Figure 7 presents, Height was negative relative to PICC associated thrombosis, the dependent variable value was assigned to 0 when running ROC software and presented by Fig. 7(a,b).

**Figure 8:**
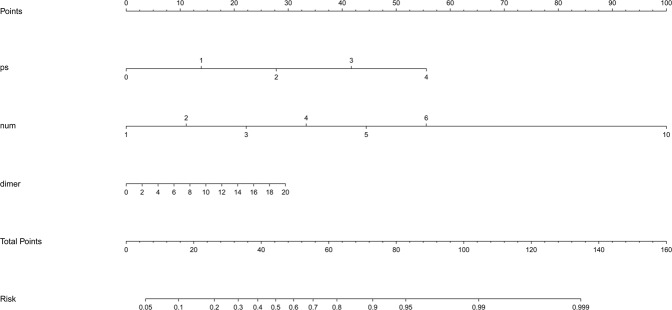
Nomogram for the risk factors of PS, Number and D-dimer.

Figure 8 presents, in terms of risk regression coefficient of PS, Number and D-dimer, the nomogram for the data visualization was made by software of R language.

Complications: Local infection in 2, catheter tip migrated to axillary vein in 2.

## Discussion

Recently, Clinical application of PICC was becoming more and more popular in cancer patients population received chemotherapy. However, There are still many problems that have been unsolved. The most concerned is PICC associated venous thrombosis. There were significant differences in the incidence of PICC associated venous thrombosis reported in different studies. Abdullah *et al*.^9^ reported that the incidence was 38.5%, while Kang *et al*.^10^ performed a retrospective cohort study of patients with lung cancer who received chemotherapy, they found that 17 out of 328 (5.2%) cases developed PICC related upper extremity venous thrombosis, with the incidence of 0.49 per 1000 catheter days. Evans RS *et al*. reported that 9% rate of asymptomatic catheter-related DVT occurred in cancer patients^11^. In our study, the overall incidence of upper extremity venous thrombosis was 17.4%, and the incidence of venous thrombosis with symptoms was 3.4%, which was consistent to the results of Kang *et al*.^10^ and Evans *et al*.^11^.

High resolution ultrasound system has been extensively applied to the assessment of upper extremity vein thrombosis. Maury^12^ proposed that US is useful for detection of late complications such as catheter related venous thrombosis, tip migration, or fibroblastic sleeve. Thrombosis in supper vena cava and catheter tip can be accurately detected by trans esophageal ultrasonography, which reduced the false negative rate, but it is unsuitable for routine application as a diagnostic tool for detecting thrombosis. The reported sensitivity and specificity for catheter-associated upper extremity thrombosis with conventional Color Doppler Flow Image (CDFI) were 100% and 93%, respectively^13^. The dependent variable (thrombosis) was observed by the way of percutaneous superficial vascular ultrasound scan, which was affected by ultrasonic wave with catheter incidence angle, vascular anatomy deviation and intra or inter observer’s variation. The superior vena cava and catheter tip can not be assessed by conventional ultrasound scan, which theoretically underestimated the incidence of PICC related thrombosis. Although based on these technical and anatomic disadvantages, because of its convenience, easy accessible, and no ion radiation, preprocedure assessment of the vascular anatomy morphology by US provides a rationale choice of the venous access. In our study, we prefer to avail ultrasound scan modality to observe PICC associated thrombosis as dependent variable before the operation and post implantation within 2 weeks.

There are three different types of thrombi observed by US. The most common type is referred to as the “fibrin sleeve”, and catheter-adherent-fibrin-sleeves thrombosis is almost always asymptomatic, but fragments of this kind of thrombosis can be detached from the catheter, which increased higher risk to pulmonary embolism (PE), however, B j j Abdullah^14^ revealed that thrombosis volume of superior extremity vein was lower than that of inferior extremity vein and symptomatic pulmonary embolism less occurred. catheter-adherent-fibrin-sleeves thrombosis can cause the inserted catheter harder to be extracted. Two otherwise types of thrombi included the mural vein thrombus and mixed thrombus^15^, in our study, the catheter adherent fibrin sleeves thrombosis were also asymptomatic.

It has also been reported that the risks of PICC related venous thrombosis were associated with multiply factors, including the proficiency and experience of the operators, puncture duration and times, catheter gauge, vein valve and anatomical variation, regular maintenance and bed-ridden time. Generally, the major causes of thrombosis were divided into blood factors, vascular factors, indwelling factors, puncture techniques, intravenous medicine properties, etc. Increased puncture times directly increased the opportunity of vascular intima injury and raised the incidence of catheter associated thrombosis. The vascular intima was injured when the catheter was implanted, and inappropriate activity by patients themselves also irritated the vascular intima, which also increased the risk of thrombosis. Repeated puncture otherwise indirectly increased the opportunity of extra-vascular stroma factor XII exposure to blood which promoted thrombosis. Therefore, it was necessary for operators to improve once-for-all puncture skill.

It was also proved that catheter related thrombosis was associated with catheter displacement, Whitman *et al*.^16^ had confirmed that the incidence of PICC related venous thrombosis was significantly decreased when the tip of the PICC catheter was located at the 1/3 of the superior vena cava. To reduce the catheter-displacement-based thrombosis incidence, the tip of catheter should be located at inferior 1/3 of the superior vena cava, which was verified by real time ECG monitoring and ensured by postoperative X-ray exam instead of Trans esophageal ultrasound. Catheter associated thrombus in 2 occurred in catheter tip migration after PICC in this study.

As vascular anatomical deviation factors. Javier E, Anaya-Ayala^17^ had reported there were three types of basilic-brachial junction anatomical variation, basilic-brachial junction at the axillary level with paired brachial veins was classified as “Type 1.” junctions assessed at the mid or lower portions of the upper arm with duplication of the brachial vein above that level were classified as “Type 2.” “Type 3.” were classified as junctions observed at the middle and lower portions of the upper arm with no duplication of the brachial vein above that level, the prevalence of variations in venous arm anatomy was Type 1: 66%; Type 2: 17%; and Type 3: 17%, respectively. Based on this study, we randomly chose the left or right upper extremity superficial vein as the observatory risk factor, no L/R limb biased. 90%, in our study, basilic vein “type 3” were selected because of relative stabilized anatomical variation as puncture point and 10% alternative brachial vein “type 2” were punctured due to easier access than basilic vein. Result suggested that the incidence was higher in left upper limb superficial vein group than that of right (*P* = 0.006), which was consistent with the result of Marnejon *et al*.^18^. Our experience indicated the right upper limb superficial vein was the preferred vein as target because of its relatively shorter and the right proximal subclavian vein entered into the cephalic trunk vein directly while the left proximal subclavian vein posteriorly lied in the sternoclavicular notch and confluent ed indirectly into the cephalic trunk vein anatomically. These physiological features may increase the difficulty in catheter placement skill, theoretically increase the incidence of catheter related thrombosis, which was with agreement of the results of Craft PS^19^. But the right limb inappropriate motion was more active than that of the left after PICC based on routine life activity, which was statistically insignificant by the multifactorial risk analysis.

As patient baseline and blood test status factors, WBC, BPC, RBC, PT, FIB, APTT, D-dimer were selected as observatory risk factors. The baseline demography, Height, Weight and BMI, BSA were included in the analysis of risk factors. Solid or Hematopoietic tumor as a independent variable and the PS score (ECOG system) also included. Furthermore, Angele and colleagues^20^ had proposed that the mechanic injury of venous, slower flow velocity and hypercoagulable state were the causes of venous thrombosis. A positive correlation was existed between whole blood viscosity, erythrocyte sedimentation, platelet aggregation and fibrinogen, which suggested that high plasma fibrinogen level could increase blood viscosity, and lead to blood in hypercoagulable state, and promote the formation of venous thrombosis^21^.

M.R. Asch^22^ and Cihan Ay^23^ had revealed tumor was a potential factor for thrombosis, tumor cells can activate the blood coagulation factor V and increase blood viscosity. Factor V represents an important control step in the clotting process. Factor V Leiden is an inherited condition, which may lead to abnormal clot formation, at common locations including lower extremity veins and at the site of indwelling plastic catheters (IVs, central lines). D-dimer concentration at high level was a indicator after the tumor resection, cancerous thrombosis and intravenous chemotherapy in the tumor patients. Nadir Y^24^ had confirmed that elevated D-dimer level was not specific for VTE and was seen in advancing age, pregnancy, post surgery and trauma, inflammatory states, infections and some cancers and their treatments. Several studies^25,26^ have also reported the D-dimer assay have a high negative predictive value and D-dimer was a sensitive but nonspecific marker of deep-vein thrombosis, however, Andreescu AC^27^ revealed that higher D-dimer concentration was associated with the risk of DVT. Therefore, coagulable status of patients should be monitored before or during PICC indwelling. the patient’s increased D-dimer concentration before the PICC, became the predictive risk factor of PICC associated thrombosis^25,26^, Our results revealed that elevated D-dimer concentration in cancer patients was closely correlated with PICC related venous thrombosis as a risk factor. the result in our study was similar with the previous studies^28–31^.

PS, reflects not only the overall condition, but the severity of the disease and bed-ridden status, in general, and the higher the PS score, the worse the homeostasis in the patient, the longer the bed-ridden time, the easier PICC related venous thrombosis.

Although the identified variables were different between the thrombosis group and the non-thrombosis group tested by Fisher Score statistically, however, it cannot be identified whether the difference was caused by the variable itself or by the sampling error, nor the correlation coefficient between the independent and the dependent, therefore, logistic regression analysis was introduced. Sharp R^32^ performed a prospective cohort study and they revealed that the catheter to vein ratio was associated with the rate of symptomatic venous thromboembolism in patients with PICC. The catheter to vein ratio as a risk factor, all patients used the same gauge catheter, we incorporated the inserted depth and the vessel-to-catheter ratio in diameter into the one risk factor in our study, which was significantly different between thrombosis and no-thrombosis group by univariate analysis but insignificantly different by multifactorial risk analysis, which indicated ratio was excluded as a risk factor of PICC associated thrombosis. There was significant difference in location between the thrombosis group and the non-thrombosis group in our study, but the variable was ruled out as an independent risk factor for PICC associated thrombosis by logistic regression analysis, which was different from the result of Verso M, Tesselaar ME, Spencer TR and Craft PS^19,33–35^.

The higher the patients’ height is, the less likely PICC related venous thrombosis is.

However, the results of height and PS are still not uniform. Therefore, more analyses are needed.

### limitations

The present study is not without limitations. (1) The sample size was relatively limited and all the data were from only one cancer center. (2) As retrospective study, the selection criteria may have introduced a selection bias, limiting the generalizability of the results. (3) Only limited factors were studied and it was still unknown how D-dimer elevation correlates with risk of thrombosis. (4) The further relative risk factors for PICC associated thrombosis should be explored. Future studies could explore additional factors that could be involved in PICC related thrombosis, such as inflammatory and molecular markers. (5) In the future, big data-based and artificial intelligence algorithm, will be developed to predict the risk of PICC related thrombus.

## Conclusions

Total incidence of thrombosis in the study was 17.4%. Most of the thrombosis were catheter-adherent-fibrin-sleeves thrombus and asymptomatic. Our study revealed that PS (performance status), number (puncture times), D-dimer and height were independent risk factors for PICC associated thrombosis, in which, PS and number were the two strongest independent risk factors in PICC associated thrombosis.

Before the PICC, the patient’s PS score should be overall evaluated, the higher score of PS, the easier PICC related thrombosis. In the process of catheter placement, skilled performance and the lowest puncture times, were the key to preventing the formation of PICC related thrombosis.

The best prediction model, D-dimer + Number as a novel co-variable with high sensitivity was validated in diagnosing PICC associated thrombosis before PICC in cancer patients. If variable number (N) or location (L) were not taken into account before catheterization, co-variable Height + PS + D-dimer (H + P + D) to predict PICC associated thrombosis should be the optimized parameters.

Our research revealed that although PS, number, D-dimer and height were independent risk factors for PICC associated thrombosis, statistics also revealed that these variables were slightly associated with PICC related thrombosis. It is safe, effective and has convenient access for cancer patients who needed long-term infusion by PICC.

## Data Availability

The data sets supporting the conclusions of this article are included within the article.
